# Time Travel Within the History of Ethnobotany

**DOI:** 10.3390/plants15030496

**Published:** 2026-02-05

**Authors:** Raivo Kalle, Renata Sõukand

**Affiliations:** 1 Estonian Literary Museum, 51003 Tartu, Estonia; 2 Biocultural Diversity Lab, Department of Environmental Sciences, Informatics & Statistics, Ca’ Foscari University of Venice, 30173 Venice, Italy

Currently, science is increasingly being influenced by artificial intelligence (AI). Although AI can be used to write content for articles quickly and conveniently, there is also a dark side: this tool is being used more and more to “produce” articles with fictitious content. Thus, what remains permanent is history: the periods when human scientists authored articles, books and articles were published in print, manuscripts were written with pen and paper, and notes were made by hand in fieldwork diaries.

We initially thought that artificial intelligence would not significantly affect ethnobotanical research, but we were mistaken. We have encountered situations, even during the review process, where the content of an article was too good to be true. When checking the original sources, it became apparent that these were empty references, as the cited articles had never been published and the information attributed to them did not exist; these spurious references were created by AI. Therefore, references that have historically appeared in print or have been preserved in manuscripts are stable and reliable—i.e., permanent—if they were archived in the pre-AI era. That is why we, as editors, are drawn to historical ethnobotany: it is based on physically existing notes that can be reinterpreted from different perspectives again and again in light of new knowledge, yielding answers to new questions that arise.

However, a historical manuscript or book on plant use is definitive, as it remains as it was originally written. Researchers who read it in the modern day can no longer question or clarify the contents. In many cases, these scarce earlier records are the only available data, requiring researchers to interpret and reconstruct the records’ contexts. To do this, it is necessary to look at broader cultural-historical sources and cultural changes, the natural conditions of the region and their transformations, and the specific features of the written language. Even then, it is always possible to err, make false assumptions, and draw erroneous conclusions. Such errors may arise from fragmented data, intentional omissions, or prior misinterpretations of the data—whether accidental [1] or deliberate [2]—that have been carried forward for decades or even centuries [3].

It was over two years ago in November 2023 when the first edition of this Special Issue was published in print [4]. At that time, the collection was fairly popular, and thus we received numerous contributions, 13 of which made it to publication. This success revealed that the topic was popular among researchers. Even as we were closing the previous Special Issue, we still received inquiries about whether it would be possible to submit manuscripts. We discussed this with the editor-in-chief of the section and he suggested creating a new Special Issue. Since history, cultural history, and plants and their use have been of interest to us for a long time, we accepted the task of editing this new Special Issue. We have now received the requisite number of contributions for publication. It brought us great pleasure to receive manuscripts from all over the world on a range of interesting topics. This Introduction will outline the submissions that deal with the earliest periods of plant use, dating from a few thousand years ago and to the mid-20th century.

The first to take us back to the distant past Huan Wu, Xiaofeng Long, and Yanfei Geng [5] from Guizhou University, College of Tea Science. They chose Guizhou Province, China, as their research area. Tea bushes have probably been grown on forest terraces in China for thousands of years. According to archeological findings, Chinese tea culture dates back 10,000 years. Initially, the leaves of *Camellia sinensis* were collected from bushes growing naturally in forests to make tea. With the beginning of cultivation, this environment also began to be reshaped, and, over its long history, it was observed that some trees and shrubs were more suitable for co-existing with tea bushes by supporting them in their growth or protecting them from many hazards. Terraces, therefore, became the most common form of cultivation for tea bushes in that region. Forest tea gardens were only defined as agroforestry systems in 2022. According to this definition, they must contain at least 10% other species (in addition to *Camellia* spp.), must be over 0.5 hectares and over 5 meters high, and the forest must be managed in a traditional way. The authors not only examined 21 ancient written sources to determine how tea forests were described, but also conducted fieldwork in eight traditional tea forests to assess which plant species grow alongside tea bushes today. The authors found that, in early sources, tea bushes were recorded as growing together with 24 species of companion plants (including 12 tree species and 7 shrub species). The data collected during their fieldwork show that tea bushes grew alongside 81 species of plants (including 40 woody plants and 10 shrub species) on terraces, but also that there were as many as 234 species around tea bushes grown in forests (including 64 tree species and 98 shrub species). Tall trees provide shade for tea bushes, enrich the soil with nitrogen, and improve the water regime. However, the literature mentions that *Albizia chinensis* (Osbeck) Merr. is necessary because it attracts flies that would otherwise harm tea bushes. Fruit trees and vegetables are also grown between tea gardens. The authors found that people were inspired by the original tea forests to find suitable companion plants for tea bushes in established tea gardens and terraces. This knowledge also aids in the transition to agroecological agriculture because the cultivation of tea bushes growing on terraces changed in the second half of the 20th century when agrochemicals began to be widely used, which polluted the soil and water, and thus alternative solutions are now being sought.

Wendy L. Applequist, who works at the Missouri Botanical Garden, takes us on an exciting journey to Africa with the most famous botanist of his time, Ibn al-Baytar (1197–1248) [6]. Ibn al-Baytar compiled the Arabic knowledge of plants from that time and supplemented it with personal experiments and observations. His most important work included over 2300 entries, in which he described the uses of 1400 substances. Applequist provides a comprehensive overview of how Ibn al-Baytar’s legacy has been recorded and studied in the past. Additionally, in the introduction to her article, she provides examples of how and by whom plant use in historical sources have been compared with modern practices. She also discusses the people known as Berbers, from whom Ibn al-Baytar acquired and recorded plant knowledge during his lifetime. Applequist relies on articles based on fieldwork conducted among Berbers in modern times, as well as reference books for comparative research. She identifies 46 plant names in Ibn al-Baytar’s work that were associated with Berbers; however, as is typical with historical sources, she was not able to identify all the names, and thus only 38 species remained in the final selection, 28 of which (or 61%) are still in use among Berbers today. Appelquist concludes that, since Berbers were largely illiterate with no written language to support the preservation of plant use, oral traditions of plant use remained uninterrupted for about 750 years. This reveals that these plants had therapeutic effects, which could become a basis for current research into the active ingredients of medicinal plants.

Next, we move to the other side of the globe to South America. Verónica S. Lema of the Institute of Anthropology of Córdoba studied *Anadenanthera colubrina* (Vell.) Brenan var. *cebil* (Griseb.), a tree native to the southern Andes, locally called *vilca* or *cebili* [7]. This tree is known throughout the Indigenous population of this mountainous region for its psychoactive properties. Archaeological evidence suggests that the seeds of *Anadenanthera* sp. have been used in pipes for psychoactive purposes for 4000 years, and this tradition continues today. Lema reviewed the prior literature and conducted fieldwork in urban and suburban markets, interviewing vendors of this plant. For shamans, inhaling powdered seeds or smoking them in a pipe was essential to achieving visions, healing, and communicating with spirits. The seeds were also used in ethnomedicine to alleviate various ailments. Placing the seeds in amulets and wearing them as jewellery was believed to protect the owner from the evil intentions of witches. Consuming the seeds as a ritual beverage was also thought to offer protection against diseases and evil spirits. In addition, the bark of this tree was used both for tanning leather and in ethnoveterinary medicine; the leaves have also been used in ethnoveterinary medicine. Since the 19th century, all parts of the tree have been used as therapeutic agents. The use of the seeds also expanded after the arrival of the Spanish. Today, this tree holds great cultural significance, and the associated cultural practices are vibrant.

We continue our travels in the Andean region. Matteo Sartori and Julia Prakofjewa from Ca’ Foscari University studied the uses of the species *Otholobium glandulosum* (L.) J.W.Grimes, native to Chile [8]. Historically, this plant was known in that region as *Culén*. The period selected for study spans from 1646 to 1810. The authors reviewed the prior literature discussing the local population’s knowledge regarding this plant and how its use was reflected in Europe. It proved to be the case that *O. glandulosum* was a popular medicinal plant among the local population and was used both to treat skin diseases and as an infusion for digestive problems. In Europe, however, this plant was mainly advertised as a recreational tea. The authors point out that, under the influence of colonization, Indigenous knowledge and the local name of this plant were pushed aside and replaced by scientific knowledge.

Next, we move to the Atlantic coast of South America, specifically to Brazil. Plant use in that region has been selected for study by Natalia Hanazaki from the Department of Ecology and Zoology at the Federal University of Santa Catarina [9]. Hanazaki structures her article as a flowing narrative, and her qualitative research shows how even the smallest coincidences and the fates of individual ordinary people can lead to significant changes. This historical adventure begins in November 1793 when a merchant ship with a crew of sixteen men set off from northern Japan to deliver goods to the central part of the country. The voyage was expected to last only a few weeks, but due to a strong storm, the ship drifted at sea for over five months, eventually reaching the Aleutian Islands, which then belonged to Russia. From there, the shipwrecked crew were taken to central Siberia, where they were detained for almost seven years. They were not sent back to Japan because, at that time, Japan had a strict policy of isolation and forbade contact with the outside world. In 1803, the Russian Tsar decided to establish relations with Japan, and four Japanese shipwrecked crew members were taken along to help smooth relations. On the way from St. Petersburg to Japan, there was only a single stop of about 45 days in southern Brazil. After unsuccessful diplomatic negotiations with the Japanese government, the four shipwrecked men were handed over to Japanese officials in April 1805. Because of Japan’s strict isolation policy, the men were then thoroughly interrogated. The records of this interrogation were the first reports and descriptions of the flora and fauna of Brazil for the Japanese people. Several books were subsequently published about the crewmen’s journey, which later influenced the emigration of Japanese people to Brazil in the early 20th century. Hanazaki provides fascinating examples of how the shipwrecked men described both the localplant species and the cultural and ecological conditions of Brazil.

The article that follows is authored by Szabolcs Kis and Attila V. Molnár [10], who work in the Department of Botany at the University of Debrecen. They reviewed the historical literature (including newspapers aimed at a wide audience) and archival sources in Hungary from 1721 to 1943 that deal with the distribution and popular use of the highly poisonous *Cicuta virosa* L. The distribution of this plant in Hungary has significantly decreased compared to the past. The folk name of this plant varies significantly in different regions of Hungary. Overall, three major folk name areas can be distinguished for this plant, but, in total, i has been known by eight folk names throughout history. New locations where this plant used to be found were also discovered in historical sources. This shows that earlier manuscripts and written sources in the national language are an important source for both ethnobotany and biogeographical data. The authors provide a comprehensive overview of the history of poisoning cases caused by this plant in Hungary. There have been many accidents, especially involving children who mistook this poisonous plant for an edible one. However, the plant has sometimes been used deliberately to poison and kill people. There have also been numerous reports in Hungary of livestock deaths caused by *C. virosa*. Its toxicity is also one of the reasons it was removed from farmland in the past and why its distribution has decreased.

Next, we arrive in the 20th century. A large team of authors, including eight scientists from Zhejiang Agricultural and Forestry University, China, led by Ke Shi and Shuai Liao from the South China Botanical Garden, studied the legacy of Scottish plant collector George Forrest (1873–1932) in China [11]. Forrest visited China seven times between 1904 and 1932. During this time, he amassed a remarkably large collection of plant specimens, which are now kept in 31 herbaria around the world. Shi and his co-authors undertook the major task of reviewing all these digital herbaria and recorded a total of 38,603 plant specimens that Forrest collected in China. These specimens belong to 233 plant families, 1395 genera, and a total of 5426 plant species. The authors organized and harmonized the disparate data by date, region, and elevation. They used this information as a source to reconstruct each of Forrest’s expeditions to China. Shi and his co-authors subsequently wrote a compelling descriptive story, which will be a significant aid to modern scholars for understanding the history of Chinese plant research at that time. Although Forrest’s achievements as a plant collector stand out in many respects, his work not only advanced botany, but also had a profound impact on the fields of horticulture, ecology, and plant conservation. He made a particularly significant contribution to the introduction of Rhododendron species to Europe. However, the authors also pay attention to the negative aspects of his work, noting that such a colonial attitude towards collecting natural resources in another country is inconsistent with today’s ethical norms. The authors also point out that Forrest, in his passion for collecting plants, did not really care about the local natural environment, as they cite the case of his cutting down a Rhododendron tree that was important to locals and taking its logs back to England.

Next, we come to the 1970s. A large team of authors, including eleven scientists from six different research institutions, led by Andrea Pieroni from the University of Gastronomic Sciences, studied the use of medicinal plants by people living in the Susa Valley in the Italian Alps [12]. The people living in this valley are heavily dependent on the tourist seasons of summer and winter (ski tourism); during other periods, there are few permanent residents living in the area. A prior comprehensive study on the use of medicinal plants in this region was conducted in 1970 (the use of 96 plant species was documented), while a comparative study was conducted in 2018 (the use of 36 plant species was documented). A comparison between periods revealed a significant decrease over time regarding the knowledge and use of medicinal plants. This can be attributed to the socio-economic, cultural, and potentially even environmental changes that have occurred over the past half century. In addition, the authors point out that, due to the current trend of functional foods, these plants can also be used in the food industry to develop regional products (e.g., seasoning salts). This could represent one solution for keeping local plant traditions alive.

The same time period—although in this instance covering an overseas territory—was studied by an international team of authors led by Renata Sõukand from Ca’ Foscari University and Andrea Pieroni [13]. Their work describes the resilience and transformation of the biocultural heritage of wild greens foraged among the Arbëreshë, an ethnolinguistic minority, in the Argolis and Corinthia regions of Greece. Compared with historical ethnobotanical records from the 1970s, this fieldwork, conducted in 2025, not only documents previously unrecorded linguistic labels, but also new plant species. However, current Arbëreshë ethnobotanical heritage has undergone profound linguistic and cultural assimilation, with the majority of folk plant names being of Greek origin and the remainder being Albanian or a hybrid. However, the tradition of using foraged plants in boiled mixed greens (*lakra* in Arbëreshë, *chorta* in Greek) remains central to local cuisine. The authors highlight the urgent need for recognizing and supporting the continuity of this minority’s remaining linguistic and ethnobotanical diversity. They suggest that the changes that appeared in the past fifty years have been driven by the ecological availability of the plants, socio-cultural transformations, and changing taste preferences.

And so we finally arrive at the 21st century. An international group of authors, including eight researchers from six institutions, led by Raivo Kalle from the Estonian Literary Museum, examined the use of medicinal plants among Karelians and Finns living in Northern Karelia, Finland [14]. This region is interesting because the majority of former settlements of one ethnic group, the Karelians, are occupied today by the Russian Federation. However, after World War II, when this border was closed, a significant number of Karelians moved from Russia to live in Finland. A total of 67 semi-structured interviews were conducted, and the current use of 31 medicinal plants was recorded. The plant names were mainly in Finnish, with only a few wild berries, one tree, and some weeds recorded in Karelian. Overall, the use of medicinal plants has significantly decreased today compared with the historical data gathered in the 19th century. Also, most knowledgeable respondents were of mixed Finnish-Karelian origin, drawing on both ancestral (and historical) knowledge. Factors such as resettlement from Russia to Finland, which resulted in the loss of contact with their traditional ancestral lands and difficulties in adapting to the new environment, may have contributed to the small number of plants reported by people with purely Karelian origin. The official health system, which did not favor the use of medicinal plants, also played a role in hindering the continuation of this tradition. Understanding all these factors could help to better preserve traditional knowledge when future decisions are made.

Saint Augustine (354 AD–430 AD), who contemplated the concepts of “long time” versus “short time” thousands of years ago, concluded, “But in what sense is that long or short, which is not? For the past, is not now; and the future, is not yet. Let us not then say, ‘it is long’; but of the past, ‘it hath been long’; and of the future, ‘it will be long.’” [15], p. 235. All our actions today are already history tomorrow. Therefore, with our data on plant uses published today or collected in archives, we point the direction to tomorrow’s scientists so that they can say “it hath been long”, just as we rely on past scientists to make decisions for the future.

## Data Availability

All information provided in the editorial is available in this publication.
